# The influence of *Hyssopus cuspidatus* Boriss extract on lipid mediators metabolism network in asthmatic mice

**DOI:** 10.3389/fphar.2023.1066643

**Published:** 2023-03-02

**Authors:** Kong Ling-Fei, Rong Xiao-Juan, Yan Pan, Qin Tuo, Zhang Xiao-Hui, Kang Yu-Tong, Cheng Bo, Su Wen-Ling, Gao Tian-Le, Tie Cai

**Affiliations:** ^1^ State key laboratory Coal resources and Safe Mining, China University of Mining and Technology-Beijing, Beijing, China; ^2^ School of Chemical and Environmental Engineering, China University of Mining and Technology-Beijing, Beijing, China; ^3^ Xinjiang Institute of Material Medica, Urumqi, China; ^4^ State Key laboratory of Natural and Biomimetic Drugs, Peking University, Beijing, China; ^5^ Institute of Materia Medica, Chinese Academy of Medical Sciences and Peking Union Medical College, Beijing, China

**Keywords:** Hyssopus cuspidatus Boriss extract (SXCF), lipid mediators (LMs), asthma, inflammation, cytokines

## Abstract

Current drugs do not provide an absolute cure or modify the course of asthma. *Hyssopus cuspidatus* Boriss extract (SXCF) has been used as Uyghur medicine for several years to treat bronchial asthma. However, very limited research has been conducted on the therapeutic mechanisms of SXCF. Disruptions in the metabolic network of lipid mediators (LMs) are closely linked to the development of asthma. Here, we explored the therapeutic mechanism of SXCF in asthma based on the metabolic network of LMs, aiming to contribute to the understanding of SXCF in asthma treatment at the molecular level. The UHPLC-MRM strategy was used for the quantitative detection of LMs in the lung tissue and in the peripheral circulatory system (serum). ELISA was used to detect IgE in serum and cytokines in BALF. The lung tissue sections were stained with H&E to observe the infiltration of inflammatory cells, and behavioural changes in mice were observed and recorded throughout the animal experiment. In contrast to the asthma group, the opposite result was observed in the SXCF groups, where the perturbed LMs metabolic network was partly restored in a dose-dependent manner with a significant elevation of anti-inflammatory metabolites, while pro-inflammatory lipids were decreased. As significant downregulation of IgE and pro-inflammatory cytokines was observed, IgE and cytokines analysis also supported the anti-inflammatory effects of SXCF. It was also noticed that SXCF treatment reduced the number of coughs and decreased the inflammatory cell infiltration around the bronchus in mice. These results suggested that SXCF has a significant ameliorative effect on ovalbumin (OVA)-induced asthma. The modulation of LMs is a possible underlying mechanism of the SXCF effects.

## Graphical Abstract

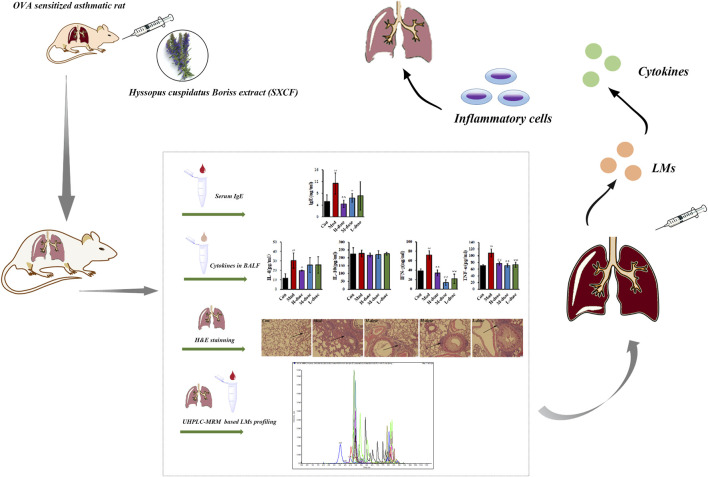

## 1 Introduction

Asthma is a common chronic airway inflammatory disease affecting over 3% of the world’s population. It causes a large number of deaths annually and poses a serious burden on global health (Aghaei et al., 2019). Alterations in the structure of the respiratory system and dysregulation of immune cells orchestrate the exaggerated chronic inflammatory process observed in the pathogenesis of asthma. (Lambrecht and Hammad, 2015). Recent treatment options are aimed at providing short-term relief of symptoms and maintaining normal lung function for a prolonged period. However, they do not completely eradicate asthma exacerbations, and their use is limited due to side effects after long-term usage (Leung, 2021). Therefore, developing drugs that are effective in preventing and treating asthma without significant side effects is an issue of great concern in the field of medicine.

*Hyssopus cuspidatus* Boriss is a traditional herb used as folk and Uyghur medicine (Yuan et al., 2019). Its chemical components include volatile oils, flavonoids, alkaloids, and organic acids. It has been used as Uyghur medicine to treat bronchial asthma (Zhao et al., 2020). Studies have shown that it inhibited the release of inflammatory cytokines (Liu et al., 2021), reduced airway hyperresponsiveness (AHR), promoted Th1, and suppressed Th2 cell functions in an ovalbumin (OVA)-induced asthma mouse model (Wei et al., 2016). The mechanism of action of SXCF is related to the pathogenesis of asthma. However, as limited studies have been conducted that elucidate the effect of this drug, we believe its development and application are still to achieve the utmost attention. Thus, its antiasthmatic action needs further assessment.

LMs are a large class of biologically active substances derived from the oxidation and metabolism of 20-carbon polyunsaturated fatty acids in the body, such as prostaglandins, hydroxy eicosatetraenoic acid (HETE), leukotrienes, epoxy fatty acids (EET), and thromboxanes (TXs) (Sheppe and Edelmann, 2021). Several lipid mediators are involved in the process of inflammation, and they develop a complete metabolic network during immune and structural cell metabolism and signalling (Sokolowska et al., 2021; Yonker et al., 2021). Moreover, it has also been demonstrated that there is a close relationship between disorders arising from the metabolism of LMs and the development and progression of asthma, manifested as lesions of the respiratory airway, blood vessels, and lung parenchyma (Su et al., 2018; Barden et al., 2021). Thus, studying the antiasthmatic mechanism of *H. cuspidatus* Boriss by targeting LMs would be of great interest. However, no in-depth study has been performed on this before, resulting in a lack of scientific data to support this theory.

In this study, LMs were used as the target, and the UHPLC-MRM strategy was used to explore the mechanism of action of SXCF in asthma, aiming at promoting the understanding of SXCF in the treatment of asthma at the molecular level and accelerating the development of drugs with antiasthmatic action.

## 2 Experiment

### 2.1 Materials

MS-grade water, acetonitrile, formic acid, acetic acid, ethanol, and methanol were purchased from Fisher Scientific (MA, United States). Isopropyl was purchased from Honeywell (UT, United States). Glycerol, OVA, and butylated hydroxytoluene (BHT) were purchased from Sigma-Aldrich (WI, United States). Deuterated LM standards (IS) were purchased from Cayman (MI, United States).

### 2.2 Preparation of medicinal materials and extracts

The herb was harvested from the Altai region of Xinjiang (identified as the dried parts of *H. cuspidatus* Boriss, by researcher He Jiang of the Institute of Pharmaceutical Sciences of Xinjiang Uygur Autonomous Region). It was crushed into a coarse powder and 1 g grounded dried herb was extracted with 12 mL 50% ethanol twice, 1.5 h each time, and the extracts were combined, concentrated under reduced pressure, and dried under vacuum to obtain the crude extract of *H.* cuspidatus Boriss. This extract was dissolved in water, sampled on polyamide resin, eluted with water, 20% ethanol, 40% ethanol, 60% ethanol, and 80% ethanol in turn. The 40% ethanol eluate was then collected, concentrated under reduced pressure, and dried under vacuum, to obtain the extract. (Batch number 20210521). The extract was analysed using UPLC-HRMS. Analytical methods and results are shown in Supplementary Material S1. SXCF UPLC-HRMS analysis.

### 2.3 Establishment of the asthma model

All animal experiments were approved by the Animal Care and Used Committee of Xinjiang Medical University as well as the Ethics Committee of the same institution (IACUC-20210603-07). After purchase, the animals were kept in an animal house at constant temperature (22°C) and 50% relative humidity, and were fed *ad libitum* and acclimatised for 1 week during the rearing period.

We randomly divided 60 BALB/C female mice with the body weight of 18–22 g into five groups; control group (A), model group (B), high-dose group (C), medium-dose group (D), and low-dose group (E) respectively. Mice from groups B, C, D, and E were fed with intraperitoneal injections of 0.2 mL OVA sensitisation solution (0.5 mg/mL in aluminium hydroxide gel saline dilution solution), while mice from group A were injected with the same amount of saline from days 1 and 8. On days 15–21, mice from group B were stimulated with atomised OVA stimulation solution (10 mg/mL OVA), once daily, 30 min each time, for 7 consecutive days, while mice from blank control group were stimulated with normal saline. Mice from groups C, D, and E were administered SXCF intragastrically (20 mg/mL, 200 mg/kg; 10 mg/mL, 100 mg/kg; 5 mg/mL, 50 mg/kg, respectively. 200 mg/kg equivalent to 4.68 g herb/kg; 100 mg/kg equivalent to 2.34 g herb/kg; 50 mg/kg equivalent to 1.17 g herb/kg). The dose was determined according to the previous study (Yuan et al., 2019). After 1 h, atomised OVA was used for stimulation, once a day, 30 min per dose, for 7 consecutive days. During the experiment, the mice had free access to food and water. Two mice in group C died.

### 2.4 Behavioural score determination

The number of sneezing and nasal scratching and the degree of asthma were recorded for each mouse after nebulisation and were scored according to the following criteria: no sneezing: 0 points, <4 times: 1 point, 4–10 times: 2 points, >11 times: 3 points. No nasal scratching: 0 points, mild nasal scratching: 1 point, frequent nasal scratching: 2 points, more than one nasal scratch: 3 points. No wheezing: 0 points, shortness of breath: 1 point, significant wheezing: 2 points, death by wheezing: 3 points.

### 2.5 Blood sample collection

We divided groups A, B, C, D, and E into two subgroups 24 h after the end of the final stimulation. Serum and lung tissue were obtained from group 1, and bronchoalveolar lavage fluid was obtained from group 2.

Serum: Samples were obtained from the eyes of mice, placed in Eppendorf tubes, left for 2 h at room temperature, centrifuged at 3000 rpm for 15 min, and stored at −20°C for IgE detection and metabolomic assay.

Lung tissues: After blood sampling, the mice were dislocated and executed, and the lung tissues were immediately removed and rinsed well with saline. The left lung was placed in a 5 mL Eppendorf tube containing 4% paraformaldehyde solution for morphological analysis; the right lung was instantly allowed to freeze in liquid nitrogen for 30 min and then transferred to a −80°C freezer for storage to ease LMs determination.

Bronchoalveolar lavage fluid (BALF): The mice were executed *via* decortication, the peritracheal tissue was immediately separated, a transverse incision was made in the upper trachea (between the third and fourth tracheal rings), a 1 mL syringe needle was inserted into the trachea (approximately 2 mm) and fixed, and the left lung was washed thrice using saline, 0.5 mL each time (80%–90% back), and the right main bronchus was ligated. The recovered BALF was centrifuged twice at 2000 rpm for 10 min, and the supernatant was divided into 200 uL/tube and stored at −80°C for the determination of cytokines.

### 2.6 Histological analysis and cytokine detection in bronchoalveolar lavage fluid and serum

The lung tissue was fixed with 4% paraformaldehyde for 48 h, dehydrated conventionally, embedded in paraffin, sectionalised, stained with haematoxylin and eosin (H&E), and the lung tissue damage was observed under a light microscope. The levels of TNF-α, IL-4, IL-10, and IFN-γ in BALF and IgE in serum were detected by ELISA purchased from Lianke (Zhejiang, China).

### 2.7 Biological sample preparation for LMs determination

We mixed 50 μL serum with 70 μL 10% glycerol solution. The solution was diluted with 25% acetonitrile solution to a final volume of 1 mL (containing 20 μg BHT and 0.5 ng IS), prior to further SPE purification.

We equally mixed 10 mg ground lung tissue with 250 μL acetonitrile. After centrifuging at 15,000 rpm for 5 min, 200 μL supernatant was transferred into a tube containing 70 μL 10% glycerol solution. The solution was diluted with 25% acetonitrile solution to a final volume of 1 mL (containing 20 μg BHT and 0.5 ng IS), prior to further SPE purification.

Sample solutions were loaded on Waters MAX SPE columns (MA, United States) initialised with acetonitrile and 25% acetonitrile, after washing with 25% acetonitrile and acetonitrile. Acetonitrile containing 1% formic acid eluent was collected and dried using a vacuum concentrator. Prior to UHPLC-MRM analysis, the dried samples were stored under −20°C.

The dried sample was reconditioned with 50 μL acetonitrile/methyl alcohol (50/50, v/v) and injected directly.

The remaining supernatant of lung tissue samples was added to 100 μL water to determine the protein content using BCA kit (Pierce, Rockford, IL, United States). The concentration of eicosanoids in lung tissue was corrected using the corresponding protein content.

### 2.8 UHPLC-MRM analysis

LMs analysis was performed on the LC-MS system consisting of a SCIEX Triple Quad 5500 + MS with an ESI source (MA, United States) and a Thermo Scientific Dionex Ultimate 3000 HPLC (MA, United States). A Waters ACQUITY UPLC BEH C18 (2.1 × 50 mm) was adopted. Mobile phase A was 0.1% acetic acid aqueous solution, and mobile phase B was acetonitrile/isopropanol (9/1 v/v). Elution conditions are shown in Table 1. The flow rate was 0.4 mL/min. The column temperature was set as 40°C. The injection volume was 5 μL. Data acquisition was performed with negative mode. The MRM condition was presented in Supplementary Table.S3 and the total ion chromatography (TIC) of LMs in lung tissue and serum samples were showed in Supplementary Figures. S3, S4.

**TABLE 1:** Gradient elution program for LM analysis.

Time/min	A%	B%
0	75	25
1	75	25
8	5	95
8.5	5	95
8.51	75	25
10	75	25

### 2.9 Data processing and statistical analysis

The data acquisition was carried out using Analyst 1.7.1 (ABsciex, MA, United States). Version 1.6 OS-Q (ABsciex, MA, United States) was employed for peak integration and quantification. IBM SPSS (Armonk, New York, United States) was employed for one-way ANOVA and Fisher’s least significant difference tests (LSD), EZinfo (Waters, MA, United States) was employed for principal component analysis (PCA) and partial least squares-discriminant analysis (PLS-DA). The opensource tool of Metaboanalyst 3.0 (HYPERLINK: http://www.metaboanalyst.ca/) was also employed for Heatmap analysis. The results are presented as mean ± standard deviation. The difference between the groups was considered significant at *p* < 0.05.

## 3 Results

### 3.1 SXCF ameliorates behavioural changes in asthmatic mice

**FIGURE 1:**
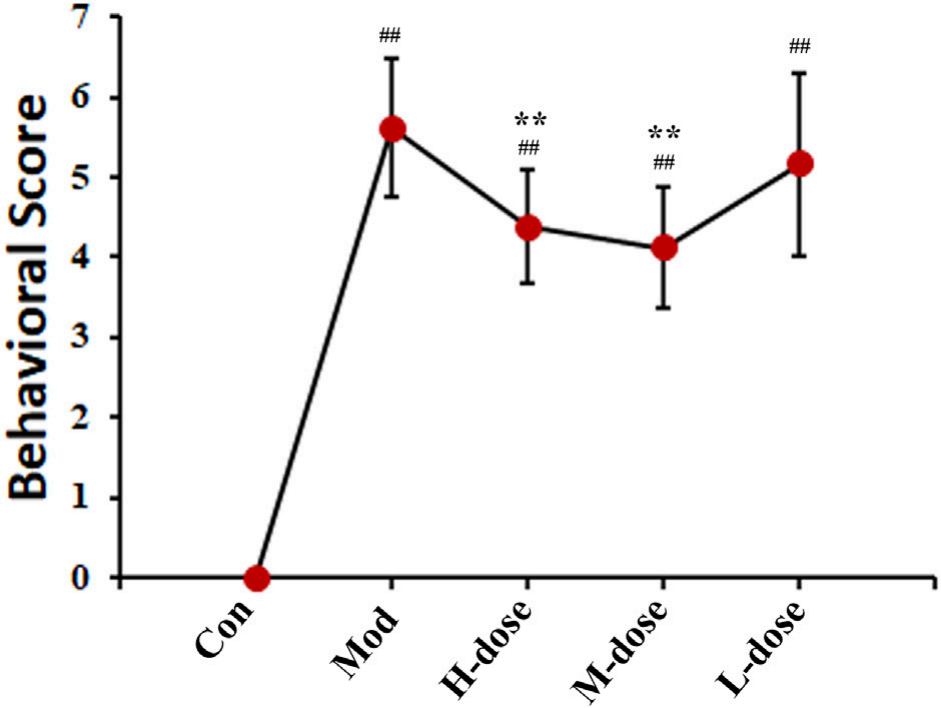
SXCF decreased the frequency of asthmatic behaviours (*n* = 6, ## vs*.* Con, *p* < 0.05, * vs*.* Mod, *p* < 0.05, data were expressed as mean ± standard deviation [one-way ANOVA followed by LSD test].).

The symptoms of asthma are usually cough, wheezing, dyspnea and changes in behaviour (Zhou et al., 2022). All groups of mice successively showed different degrees of head and facial itching, irritability, limb licking, head nodding, and breathing. The behavioural changes in each group were scored and the behavioural characteristics of the mice were statistically analysed according to the number of sneezes, nose scratches, and panting, as shown in Figure 1. The behavioural scores of the OVA-induced model group were significantly higher than those of the control group, indicating that OVA successfully induced asthma in mice. The scores of mice treated with SXCF were significantly lower than those of mice in the asthma group, indicating that SXCF could significantly improve the symptoms of OVA-induced asthma in mice.

### 3.2 SXCF attenuates IgE secretion in the serum as well as pro-inflammatory cytokines in the bronchoalveolar lavage fluid

**FIGURE 2:**
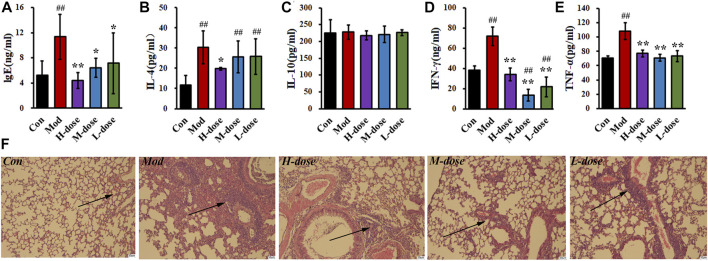
SXCF reduces the expression of relevant cytokines in serum and BALF and decreases the infiltration of inflammatory cells. **(A)** IgE in serum, **(B)** IL-4 in BALF, **(C)** IL-10 in BALF, **(D)** IFN-γ in BALF, **(E)**. TNF-α in BALF. **(F)**. Lung histology analysis of different groups. n = 6, ## vs*.* Con, *p* ≤ 0.01, ** vs*.* Mod, *p* ≤ 0.01, * 0.01 < *p* < 0.05, data were expressed as mean ± standard deviation (one-way ANOVA followed by LSD test).

The serum IgE level in the asthma group was twice that in the control group, as shown in Figure 2, indicating that the mice asthmatic model was induced by OVA sensitisation. With SXCF treatment, a dose-depended reverse regulation was observed. Thus, SXCF possibly inhibited IgE synthesis in asthma mice, thereby proving a potential to relieve the symptoms of mice by decreasing IgE levels.

The levels of cytokines associated with inflammation and immune processes of BALF were measured. As shown in Figures 2B–E, significant elevating levels of cytokines in the BALF were induced by the OVA challenge. Compared with the control group, IFN-γ and TNF-α increased by 100%, and IL-4 increased by over 200%. After SXCF treatment, those elevating concentrations were mainly reversed. TNF-α was downregulated to 70 pg/mL, similar to that of the control group. IFN-γ decreased below 40 pg/mL with a high-dose SXCF. IL-4 was observed to decrease significantly with a high dose of SXCF.

### 3.3 SXCF ameliorates lung injury

H&E staining was used to detect pathological changes in the lung tissue of mice, as shown in Figure 2F. OVA sensitisation resulted in inflammatory cell infiltration around bronchus, as well as bronchial walls thickening. Lung injury was levelled based on the cell infiltration and morphological changes as shown in Supplementary Material S2. With SXCF treatment, the lung injury was reversed associated with dosages. In high-dose group, 4 mice (67%) were detected with minor lung injury, the rest 2 mice (33%) were with moderate lung injury. With decreasing dosage, 3 mice (50%) were detected with minor lung injury both in medium-dose and low-dose groups. Notably, 1 mouse (17%) in the L-dose group was with severe lung injury.

### 3.4 Both LMs in the lung and serum were affected by SXCF treatment

**FIGURE 3:**
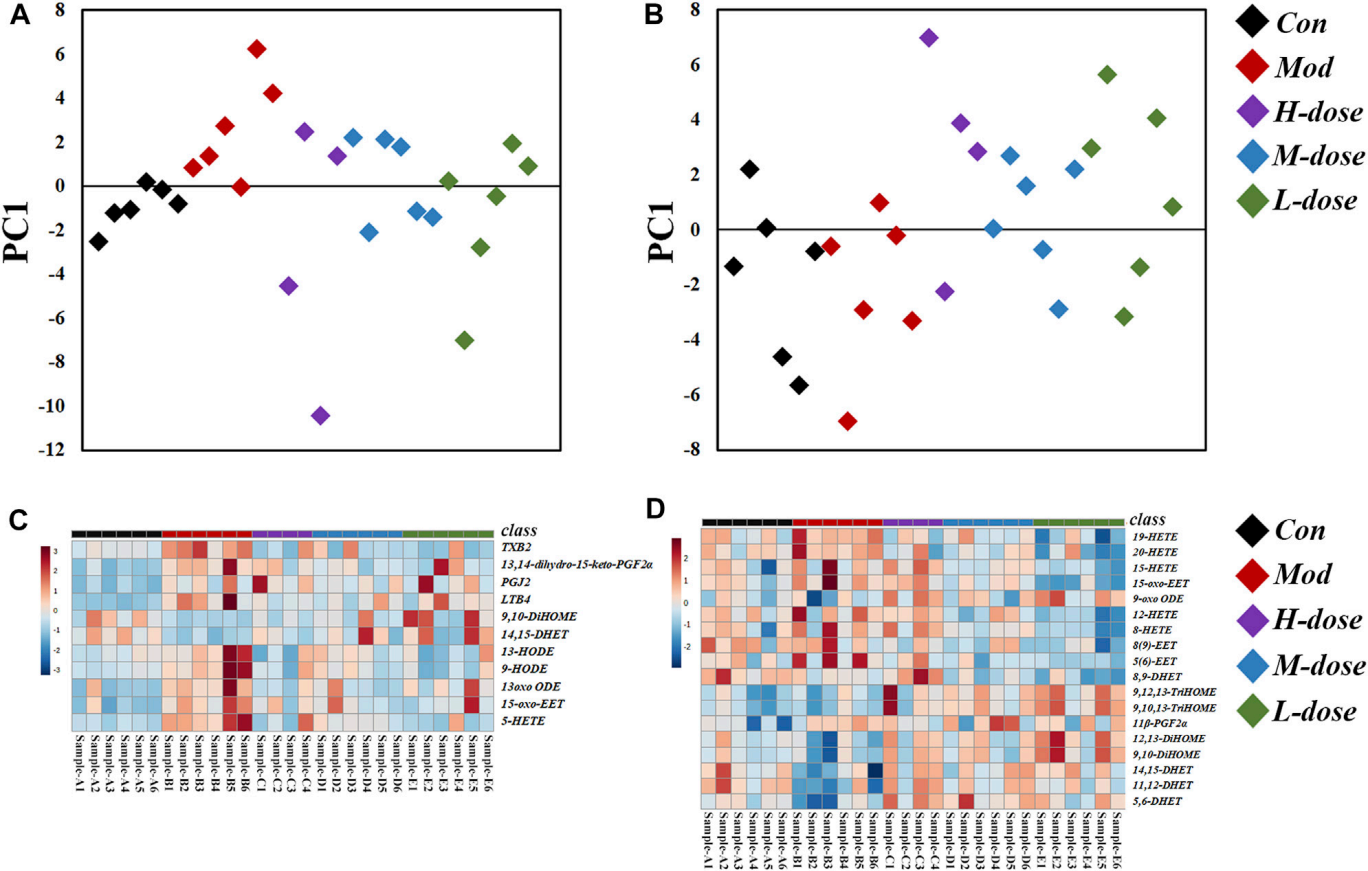
LM regulation of OVA-induced asthma group and the control group. **(A)** PCA score scatter plot of lung tissue. **(B)** PCA score scatter plot of serum. **(C)** Heatmap of lung tissue. **(D)** Heatmap of serum.

In lung tissue, after OVA challenge, the scatter plot based on the principal component analysis (PCA) scores indicated a clear separation of levels of LMs between the control and asthma group (Figure 3A). PCA showed that the levels of LMs of OVA-induced mice treated with SXCF was significantly different from that of untreated OVA-induced mice. Reverse regulation of LMs concentration induced by the SXCF was observed in the three treated groups and close to that of the control group, which indicates that SXCF was able to normalize the LMs metabolic profile. In serum, there was little overall change of LMs after OVA challenge (Figure 3B). It was believed that LMs released from the lungs were diluted by serum sharply. Any LM changes levelled to serum background were not easily detected. Those trends were also found in heatmap, as shown in Figures 3C,D.

### 3.5 Specific changes of LMs between SXCF treatment groups

**FIGURE 4:**
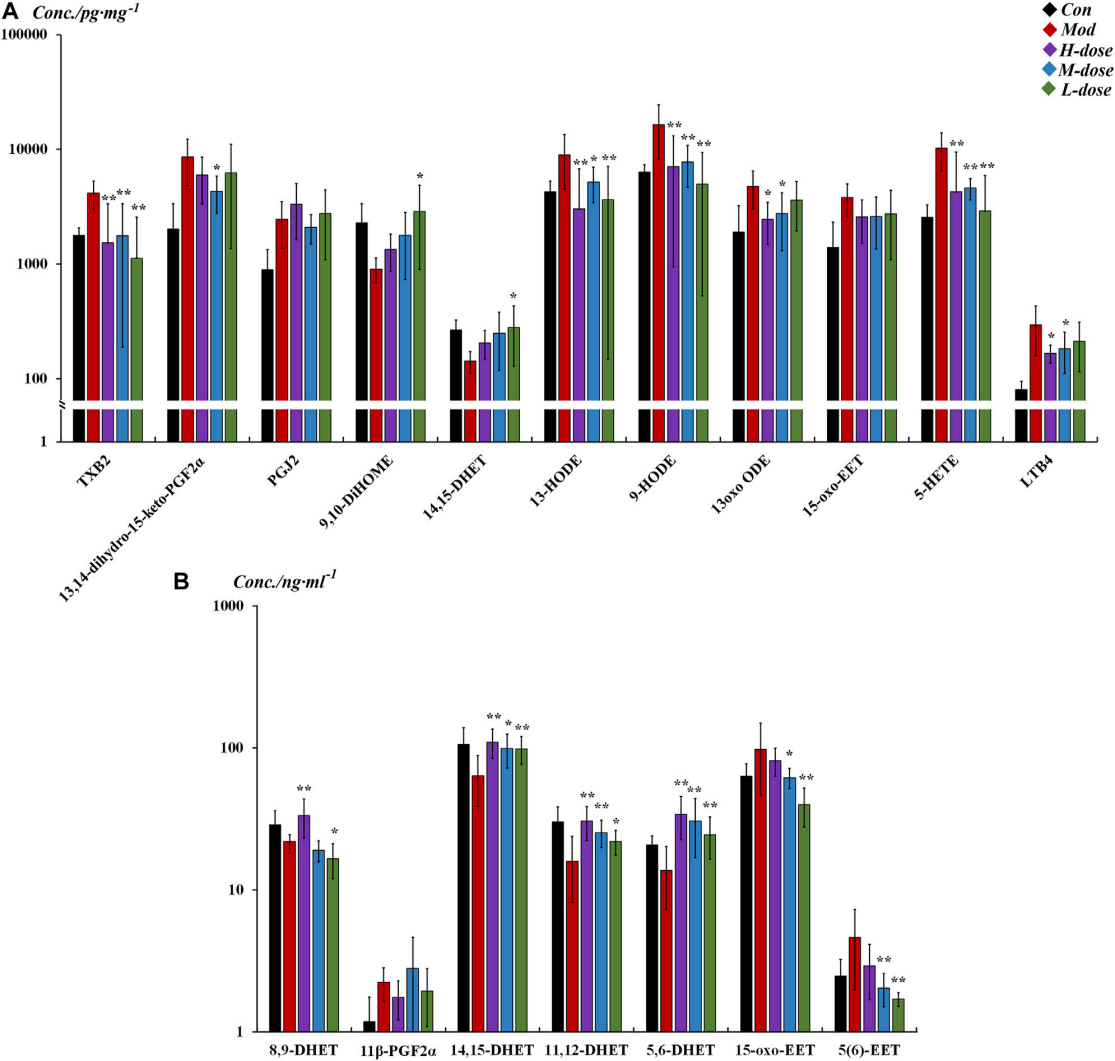
Different LM regulation capabilities between different groups in lung tissue and serum (All listed LMs were found to be significantly changed [*p* < 0.05] in tissue between control and model groups, with SXCF treatment, some of these LMs were found to be significantly changed [*p* < 0.05]). **(A)** LMs with significant changes in lung tissue, **(B)** LMs with significant changes in serum. n = 6, ** vs*.* Mod, *p* ≤ 0.01, *0.01 < *p* < 0.05, data were expressed as mean ± standard deviation (one-way ANOVA followed by LSD test).

**FIGURE 5:**
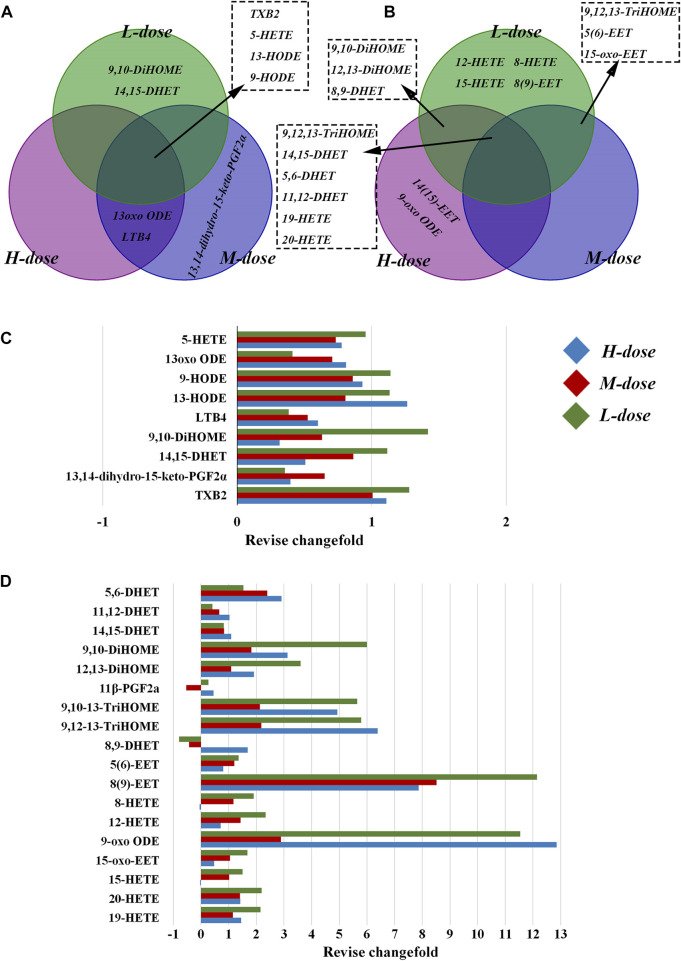
Differential regulation of LMs by different doses of SXCF. **(A)** LMs regulated by different doses of SXCF in lung tissue, **(B)** LMs regulated by different doses of SXCF in serum, **(C)** Fold-change of alteration in lung tissue, **(D)** Fold-change of alteration in serum.

As shown in Figure 4, in the lung tissue, 11 LMs with differential changes after OVA challenge were identified, and some were regulated by SXCF through a dose-dependent manner, such as 13 oxo ODE and LTB4. High-dose SXCF treatment reversed the concentration changes of 6 LMs, accounting for 66.7% of 11, while the medium-dose SXCF treatment for 77.8% (7 of 11) of the LMs, and the low-dose SXCF treatment for 66.7% (6 of 11) of the LMs. Figure 5A demonstrates that the regulated LMs species overlapped in three dosage groups. In lung tissue, 4 LMs species were found to change significantly in all three dosage groups. Although the regulated LMs species overlapped in three dosage groups, the concentrations of LMs were differently associated with dosage. As shown in Figure 5C, the change-folds of regulation in the medium-dose group were all within 1, reasonably within the range of variation, indicating that the medium-dose SXCF intervention did not over-regulate the relevant LMs. However, the high-dose and low-dose interventions resulted in a fraction of LMs with a modulation multiplier exceeding 1 and even reaching 1.5, especially in the low-dose group. Thus, the administration of high and low doses probably led to a compensatory response.

In serum, although only 7 differentially altered LMs were found after OVA sensitisation, 16 differentially altered LMs were found in the low-dose group, even though there were relatively fewer in the high and medium-dose groups, with 11 and 9 LMs, respectively (Figure 5B). These LMs were not only “off-target,” but most also possessed large change-folds, as shown in Figure 5D.

**FIGURE 6:**
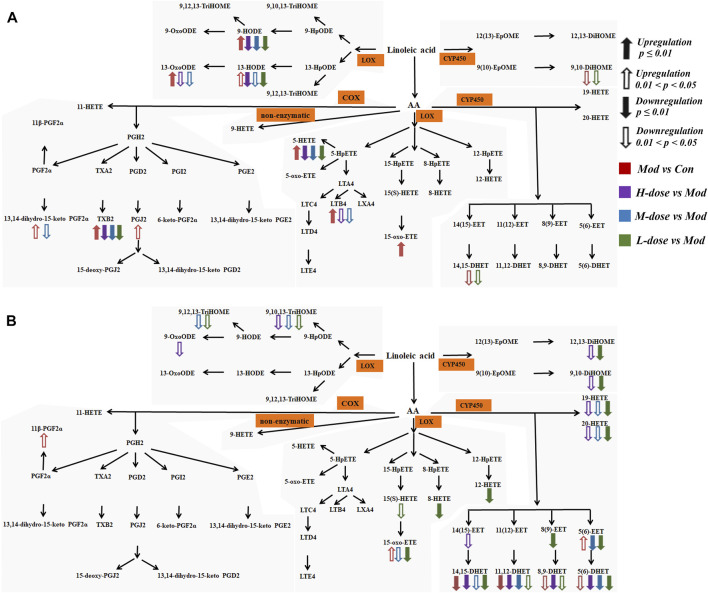
LMs detected in biological samples with their metabolism pathway and variations among different groups. **(A)** LMs in lung tissue. **(B)** LMs in serum.

The significantly altered LMs were almost diametrically opposed in lung tissue and serum, as shown in Figure 6. In the lung tissue, the effects of SXCF on LMs metabolic network were mainly restricted in upstream metabolites of Linoleic acid (LA) metabolic pathway, COX-catalysed Arachidonic acid (AA) metabolic pathway, and 5-LOX-catalysed AA metabolic pathway. In serum, the effects of SXCF were mainly restricted to downstream metabolites of LA metabolic pathway, AA metabolic pathways catalysed by 15-LOX and 12-LOX, and AA metabolic pathways catalysed by CYP450 enzymes.

### 3.6 TXB2, 5-HETE, and HODEs are potential biomarkers for SXCF treatment of asthma

**FIGURE 7:**
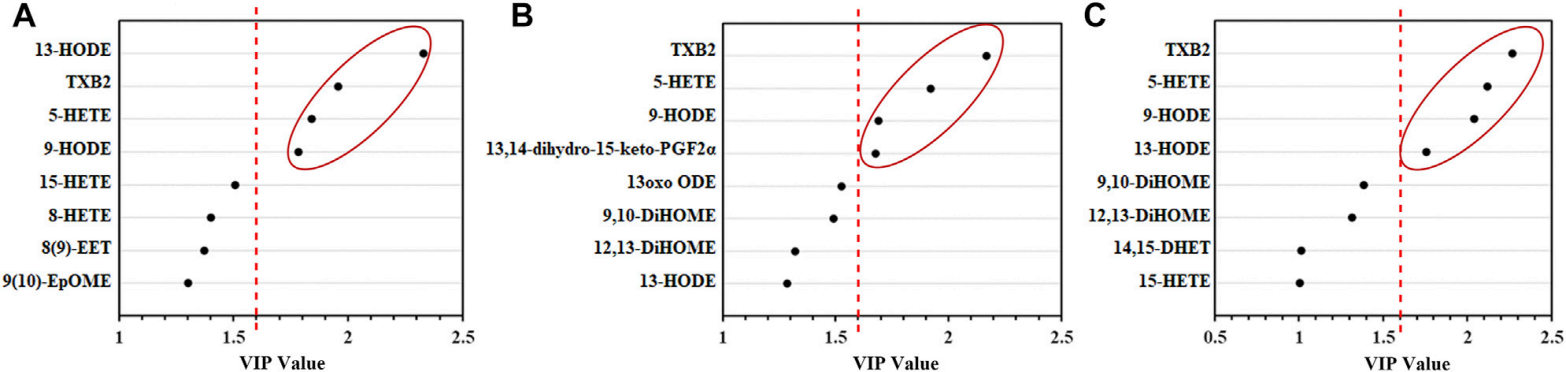
Results of PLS-DA analysis of lung tissue, the right side of the red-dashed line shows the difference LMs with VIP values > 1.6. **(A)** High-dose group **(B)**. Medium-dose group **(C)**. Low-dose group. n = 6.

Based on the VIP score of PLS-DA for the change in concentration of LMs in the SXCF treatment group (Figure 7), combined with the results of one-way ANOVA and LSD test, LMs meeting VIP>1.6 and *p* < 0.05 were screened. TXB2, 5-HETE, and HODEs were the potential biomarkers for SXCF treatment of asthma were identified.

## 4 Discussion

Current antiasthmatic drugs have a single target and cause systemic adverse effects after prolonged usage, a situation that has led to a great number of patients suffering from asthma with unfavourable disease control. Since asthma is an allergic disease involving several targets, there is a risk of losing key information through pharmacological studies that involve a single target. Many LMs are critically involved in the induction and regression of inflammation, and they develop a complete and well-established metabolic network that is effectively involved in immune and structural cell metabolism and signalling (Cai et al., 2019). We used the complete LM metabolic network as the therapeutic target of SXCF and identified the key metabolites closely associated with SXCF in the treatment of asthma. Based on this, we investigated the efficacy and mechanism of SXCF in allergic asthma by combining inflammatory factors such as TNF-α, IL-4, IL-10, IFN-γ, serological indicators, behavioural results, and pathological results.

Serum IgE is elevated in majority of patients with asthma. It is critically involved in the development of allergic airway inflammation and airway hyperresponsiveness. It is a known biomarker for asthma (Krause et al., 2020). As reported in previous studies (Kim et al., 2012; Ma et al., 2016), TNF-α is the promoter of IgE production. TNF-α increases IL-4 and IL-10 expression and upregulates IgE consequently. IL-4 promotes IgE production by B cells and exacerbates inflammatory cell infiltration (Li et al., 2021). After treatment with SXCF, some cytokines were reversed in a dose-dependent manner, with most in the high-dose group similar to that of the control group. TNF-α and IFN-γ are also known to be involved in the chemotaxis of inflammatory cells (Chong et al., 2006). Apparently, in parallel with the increase of TNF-α and IFN-γ, many inflammatory cells infiltrated the bronchi and perivascular areas in the asthma model group, with inflammatory cell exudation in the bronchial lumen, bronchial epithelial degeneration, and mucosal thickening. After administering SXCF, the aforementioned pathological conditions were alleviated, and the number of inflammatory cells was reduced. Therefore, our findings revealed that SXCF has a protective effect against airway inflammation induced by OVA in the mice model. Thus, experimentally confirming its therapeutic use as reported previously (Wei et al., 2016; Yuan et al., 2019).

LMs can perform various functions in the inflammatory process of asthma, either directly or through the action of their metabolites. As suggested, 20-HETE plays a key role in mediating acute ozone-induced AHR (Cooper et al., 2010). It has also been reported that 20-HETE appears to be a prostaglandin H2/TXA2 receptor, thereby inhibiting thromboxane biosynthesis (Hill et al., 1992). Further, it has been observed that EETs are involved in the regulation of airway mucosal fluid composition and lung inflammation (Jacobs and Zeldin, 2001; Smith et al., 2005). The metabolites of linoleic acid 9 (10)-EpOME and 12 (13)-EpOME and DiHOME are commonly referred to as leukotoxins (Le Quéré et al., 2004). Several animal studies have shown that acute lung injury and neutrophil infiltration are associated with leukotoxin injection (Totani et al., 2000). After SXCF treatment, most of these LMs concentration changes were reversed, which might suggest that SXCF can exert antiasthmatic effects by modulating multiple lipid mediator targets in the lungs and serum. The results of characterization of the metabolic network of LMs in lung tissue were matched to IgE in serum and cytokine in BALF, and it was believed that multiple LMs modulated by SXCF exert synergistic effects (Powell, 2021), exhibiting a partially dose-dependent pharmacological effect. However, the significantly altered LMs were nearly diametrically opposed in lung tissue and serum. This could be due to compensatory metabolism in other organs. Compensation is a self-protective mechanism in diseases, leading to a state of homeostasis that deviates from that in physiological conditions (Fan et al., 2011). After the abnormally regulated compounds in the organ (lung tissue) enter the blood, they may affect the response of other tissues and organs (Adib-Conquy and Cavaillon, 2009), such that these abnormal LMs increase their degradation and metabolism. Therefore, alterations of LMs concentration in serum may be the result of compensatory metabolism in other tissues and organs.

Furthermore, our findings revealed that TXB2, 5-HETE, and HODEs were critical in the treatment of asthma using SXCF. Thromboxane A2 (TXA2) is a potent smooth muscle constrictor that induces bronchoconstriction with a half-life of approximately 30 s. It is then metabolised to the inactive and stable TXB2, such that TXB2 levels can represent the TXA2 levels (Shi et al., 2022). It is known that antigen-induced airway hyperreactivity is accompanied by a significant increase in TXA2 and infiltration of granulocytes, which cause bronchial obstruction *via* IgE secretion (Ramos-Ramírez et al., 2013). As reported, 5-HETE induced mucus secretion, airway constriction, and chemotaxis of neutrophils (Bittleman and Casale, 1995; Schultz et al., 2020). TXA2 and 5-HETE were significantly increased in the asthma group. Excess TXA2 and IgE led to severe bronchial obstruction (Davila et al., 2015), and the excess of 5-HETE caused airway constriction and increased mucus, leading to sneezing and rapid breathing during the excitation period and inducing many granulocytes to accumulate around the lung tissue (Schultz et al., 2020), which manifested as a significant infiltration of inflammatory cells in the model. Prior to our study, several studies have used TXB2 and 5-HETE as biomarkers of airway inflammatory diseases. Takaku et al. (2016). detected stable TXB2 in exhaled breath condensate of asthma patients. Zhou et al. also reported that TXB2 is in some way associated with airway inflammation (Zhou et al., 2018). Similarly, Cai et al. (2019) suggested that HETEs metabolized from HPETE *via* lipoxygenase (LOX) should be a potential biomarker to distinguish ACO from COPD. Moreover, HODEs also have pro-inflammatory effects, and their most important targets are macrophages and monocytes (Vangaveti et al., 2010; Rolin et al., 2014). In the pathogenesis of asthma, the amount of HODEs increases, and a large volume of HODEs acts on macrophages and monocytes. The activated monocytes can then produce and release various cytokines (Filion et al., 2003), such as interferon and tumour necrosis factor, due to which we can detect significant changes in TNF-α, and IFN-γ in BALF. SXCF treatment significantly reversed the abnormal upregulation of LMs induced by the asthma model, thereby reversing the changes of cytokines such as TNF-α and IL-4. This, in turn, reduced the degree of inflammatory cell infiltration and ultimately achieved the reduction of asthma symptoms.

## 5 Conclusion

Our findings revealed that SXCF has beneficial effects on the regression of asthma symptoms. The levels of IgE and pro-inflammatory cytokines were significantly downregulated, and the number of coughs and inflammatory cell infiltration around the bronchus in mice were decreased. These changes may be caused by the regulation of key LMs (TXB2, 5-HETE, and HODEs). SXCF treatment significantly reversed the abnormal changes of these LMs concentration, thereby alleviating the symptoms in these mice. This sheds more light and understanding on the mechanism of SXCF in the treatment of asthma at the molecular level and provides new possibilities for further development of drugs with antiasthmatic action.

## Data Availability

The raw data supporting the conclusion of this article will be made available by the authors, without undue reservation.

## Abbreviations

5-HETE, 5-Hydroxyeicosatetraenoic acid; AA, Arachidonic acid; BALF, Bronchoalveolar lavage fluid; BCA, Bicinchonininc acid; BHT, Butylated hydroxytoluene; DiHOMEs, solutioninmethylacetate; HE, Hematoxylin and eosin; HODEs, hydroxyoctadecadienoic acids; IFN, Interferon; IgE, Immunoglobulin E; IL, Interleukin; LMs, Lipid mediators; LTs, leukotrienes; LXs, Lipoxins; OVA, Ovalbumin; PCA, Principal Component Analysis; PLS-DA, Partial least squares-discriminant analysis; SPE, Solid-Phase Extraction; SXCF, Hyssopus cuspidatus Boriss extract; TXA2, thromboxane A2; TNF, Tumor Necrosis Factor.
