# Supplementary material for: The influence of Hyssopus cuspidatus Boriss extract on lipid mediators metabolism network in asthmatic mice
Source: Front Pharmacol. 2023 Mar 2;14:1066643. doi: 10.3389/fphar.2023.1066643 (PMC10017864; doi:10.3389/fphar.2023.1066643)
Supplement: Supplementary file 1 [file DataSheet1.docx]

**The influence of *Hyssopus cuspidatus* Boriss Extract on Lipid Mediators Metabolism Network in Asthmatic Mice**

**Kong Ling-Fei^1,2†^, Rong Xiao-Juan^3*†^, Yan Pan^1,2^, Qin Tuo^1,2^, Zhang Xiao-Hui^4^, Kang Yu-Tong^3^,** **Cheng Bo^3^,** **Su Wen-Ling^3^, Gao Tian-Le^5^, Tie Cai^1,2**^**

^1^State key laboratory Coal resources and Safe Mining, China University of Mining and Technology-Beijing, Ding11 Xueyuan Road, Beijing 100083, China

^2^School of Chemical and Environmental Engineering, China University of Mining and Technology-Beijing, Ding11 Xueyuan Road, Beijing 100083, China

^3^XinJiang Institute of Material Medica, South Xinhua Road 140, Urumqi 830004, China

^4^State Key laboratory of Natural and Biomimetic Drugs, Peking University, 38 Xueyuan Road, Beijing 100191, China

^5^Institute of Materia Medica, Chinese Academy of Medical Sciences & Peking Union Medical College, 1 Xian Nong Tan Street, Beijing 100050, China

† These authors contributed equally to this work and share first authorship

**Corresponding Author**

* Rong Xiao-Juan, 109303620@qq.com

** Tie Cai, [tiecai@cumtb.edu.cn](mailto:tiecai@cumtb.edu.cn), 86-10-62339522

**1. SXCF UPLC-HRMS analysis**

SXCF was diluted with 50% methanol and analyzed by Waters UPLC-HRMS system (H-Class UPLC-Synapt G2-Si HDMS, MA). A Waters ACQUITY UPLC BEH C18 (2.1×50 mm) was adapted. Mobile phase A was 0.1% formic acid aqueous solution, and mobile phase B was 0.1% formic acid acetonitrile solution. Elution conditions are shown in Table 1. The flow rate was 0.3 mL/min. The column temperature was set as 30°C. The injection volume was 2 μL. MS data acquisition was performed with negative-mode and positive-mode.

| **Table s1. Gradient Elution program for SXCF UPLC-HRMS analysis** | | |
| --- | --- | --- |
| **Time/min** | **A%** | **B%** |
| 0 | 100 | 0 |
| 2 | 100 | 0 |
| 10 | 90 | 10 |
| 28 | 0 | 100 |
| 30 | 0 | 100 |
| 30.1 | 100 | 0 |

**Figure s1.** TIC of SXCF (negative-mode).

**Figure s2.** TIC of SXCF (positive-mode).

**2. Analysis of pathological findings of lung injury in mice**

Combined with the pathological histological changes of the lung in this test, the degree of lung injury was graded by using bronchial and perivascular inflammatory cell infiltration, bronchial obstruction, and interstitial inflammatory cell infiltration in the lung as the main observation indicators.

"-" Normal lung tissue morphology, no abnormal changes were observed.

"+" Minor (mild) pathological changes in a few parts of lung tissues (bronchi, interstitial lung).

"++" Moderate (moderate) pathological changes in some lung tissues (bronchi, interstitial lung).

"++++" Significant (severe) pathological changes in most lung tissues (bronchi, interstitial lung).

| **Table s2. Analysis of pathological findings of lung injury in mice** | | | | |
| --- | --- | --- | --- | --- |
| **Groups (n=6)** | **Classification of the degree of lung injury** | | | |
|  | — | + | ++ | +++ |
| Con | 6 | 0 | 0 | 0 |
| Mod | 0 | 0 | 2 | 4 |
| H-dose | 0 | 4 | 2 | 0 |
| M-dose | 0 | 3 | 3 | 0 |
| L-dose | 0 | 3 | 2 | 1 |

| **Table s3. MRM Condition** | | | |
| --- | --- | --- | --- |
| ID | Q1 Mass(Da) | Q3 Mass(Da) | CE(volts) |
| 6-keto-PGF1a | 369.3 | 163.2 | -46 |
| TXB2 | 369.2 | 169.1 | -35 |
| 9,12-13-TriHOME | 329.2 | 211.1 | -38 |
| 9,10-13-TriHOME | 329.2 | 171.1 | -42 |
| PGF2a | 353.2 | 309.3 | -38 |
| PGE2 | 351.2 | 271.301 | -38 |
| PGD2 | 351.2 | 271.3 | -36 |
| Lipoxin A4 | 351.2 | 115.2 | -30 |
| PGB2 | 333.2 | 235.3 | -38 |
| LTB4 | 335.2 | 195.1 | -33 |
| 12,13-DHOME | 313.2 | 183.2 | -42 |
| 9,10-DHOME | 313.2 | 201.2 | -40 |
| 14,15-DHET | 337.2 | 207.1 | -34 |
| 11,12-DHET | 337.2 | 167.1 | -38 |
| 8,9-DHET | 337.2 | 127.1 | -40 |
| 15-deoxy PGJ2 | 315.2 | 271.3 | -30 |
| 19-HETE | 319.2 | 275.1 | -34 |
| 20-HETE | 319.2 | 275.2 | -33 |
| 5,6-DHET | 337.2 | 145.1 | -36 |
| 13-HODE | 295.2 | 195 | -35 |
| 9-HODE | 295.2 | 171 | -35 |
| 15-HETE | 319.2 | 301.4 | -28 |
| 13oxo ODE | 293.2 | 113 | -39 |
| 11-HETE | 319.2 | 167.2 | -33 |
| 15-oxo-EET | 317.2 | 113.1 | -34 |
| 9-oxo ODE | 293.2 | 185.1 | -38 |
| 12HETE | 319.2 | 179.2 | -30 |
| 8-HETE | 319.2 | 301.2 | -27 |
| 9-HETE | 319.2 | 123.1 | -30 |
| 5-HETE | 319.2 | 115.1 | -31 |
| 12(13)-EpOME | 295.2 | 195.2 | -30 |
| 14(15)-EET | 319.2 | 219.3 | -28 |
| 9(10)-EpOME | 295.2 | 171.1 | -32 |
| 11(12)-EET | 319.2 | 167 | -30 |
| 5-oxo-EET | 317.2 | 273.2 | -30 |
| 8(9)-EET | 319.2 | 123 | -30 |
| 5(6)-EET | 319.2 | 191 | -30 |
| 11β-PGF2α-d4 | 357 | 197 | -35 |
| 13,14-dihydro-15-keto-PGF2α-d4 | 357 | 187 | -35 |
| 13,14-dihydro-15-keto-PGE2-d4 | 355 | 113 | -35 |
| 13,14-dihydro-15-keto-PGD2-d4 | 355 | 211 | -35 |
| 15-deoxy PGJ2-d4 | 319 | 203 | -35 |
| 11β-PGF2α | 353.2 | 193.2 | -35 |
| 13,14-dihydro-15-keto-PGF2α | 353.2 | 183.1 | -35 |
| 13,14-dihydro-15-keto-PGE2 | 351.2 | 175.2 | -35 |
| 13,14-dihydro-15-keto-PGD2 | 351.201 | 175.3 | -35 |
| 10,17-DiHDoHE | 359.2 | 153.2 | -35 |
| 19,20-DIHDoHE | 361.2 | 273.3 | -30 |
| LTD4 | 495.2 | 177.1 | -35 |
| Resollvin D1 | 375.2 | 141 | -35 |
| LTE4 | 438.2 | 333.2 | -30 |

**Figure s3.** TIC of LMs in lung tissue.

**Figure s4.** TIC of LMs in serum.
